# Antidepressant-like effects of the aqueous lyophilizate of the stems and leaves of *Momordica foetida* (Cucurbitaceae) in rats

**DOI:** 10.1016/j.ibneur.2025.03.002

**Published:** 2025-03-12

**Authors:** Bibiane Tatiana Diebo Kom, Gwladys Temkou Ngoupaye, Francis Bray Yassi, Aurelien Fossueh Foutsop, Blesdel Maxwell Adassi, Brunel Steve Ngoufack, Elisabeth Ngo Bum

**Affiliations:** a Department of Biological sciences, Faculty of Science, University of Maroua, Maroua, Cameroon; b Department of Animal Biology, Animal Physiology and Phytopharmacology Research Unit, University of Dschang, Dschang, Cameroon; c Department of Biological Sciences, Faculty of Science, University of Ngaoundéré, Ngaoundéré, Cameroon

**Keywords:** Depression, *M. foetida*, Monoamines, Chronic restriction, Oxidative stress

## Abstract

*M. foetida* (Cucurbitaceae) is a perennial climbing herb, known in traditional medicine for the treatment of certain diseases, such as malaria, headaches, skin-related problems and many others. The objective of this work was to evaluate the antidepressant effect of the aqueous lyophilisate of the mixture of leaves and stem of *M. foetida*. The antidepressant effect of the aqueous lyophilisate of *M. foetida* at different doses (25 mg/kg, 50 mg/kg and 75 mg/kg) was evaluated in Wistar rats of both sexes submitted to chronic restriction for 14 days, using the forced swimming test, open field test and sucrose preference test. One hour following the last behavioural test, animals were sacrificed and their hippocampi were collected for biochemical assessment of oxidative parameters, including malondialdehyde (MDA), reduced Glutathione (GSH), Catalase activity, superoxide dismutase (SOD) and nitric oxide (NO) as well as monoamines levels including serotonin, noradrenaline and dopamine. The aqueous lyophilisate of *M. foetida* significantly decreased the immobility time and significantly increased sucrose consumption (P < 0.001), with no alteration of locomotor activity. The aqueous lyophilisate of *M. foetida* significantly increased the concentrations of GSH, SOD, as well as catalase activity, while reducing the concentrations of MDA and NO at all doses (P < 0.001). *M. foetida* at the doses 25 mg/kg and 50 mg/kg significantly increased the concentration of serotonin and dopamine. Only the dose 75 mg/kg significantly increased the concentration of noradrenaline (p < 0.001). These results suggest that *M. foetida* exerts antidepressant-like effects through the modulation of oxidative stress and monoaminergic pathways.

## Introduction

Major depressive disorder (MDD) is a serious illness with a significant social, psychological and clinical impact (Bondy, 2002). Depression affects almost all aspects of a person's life, including thinking, emotions, physical health and social relationships (WHO, 2017). MDD is the leading cause of morbidity and disability in the world; according to the WHO, over 350 million people worldwide are currently living with this condition (Bondy, 2002). In Africa, its prevalence is estimated at 9 % in the general population (Endomba et al. 2020). In Cameroon, MDD is a significant public health problem. For example, in Douala, the economic capital of Cameroon, the prevalence of depressive disorders among patients recorded by general practitioners was 22.5 % for minor depressive disorders and 10 % major depressive disorders (Toguem et al. 2019). Exposure to stress from certain life events has been identified as a risk factor for the development of several neuropsychological disorders such as MDD (Lee et al. 2020).

The contribution of oxidative stress (OS) to the pathogenesis of major depressive disorder (MDD) is firmly established, largely due to the brain's increased susceptibility to oxidative damage. This vulnerability arises from its high oxygen consumption, elevated lipid content, and relatively weak antioxidant defences (Bhatt et al. 2020). Indeed, overproduction of reactive oxygen species leads to disruption of the phospholipid-rich neuronal membrane affecting the functions of serotonin and catecholaminergic receptors (Ali et al. 2021). Of particular interest, the monoamine system, predominantly serotonin and dopamine signalling, has been extensively studied and found to play a significant role in the etiology of major depressive disorder (MDD) (Moore et al. 2018).

Despite significant progress in therapeutic approaches such as psychotherapy and pharmacological therapies, notably the existence of antidepressant medication, many patients do not experience symptomatic remission or response to treatment, even after treatments with several medications (Mora et al. 2018). This is largely attributed to factors such as the development of treatment resistance relapse (Bhatt et al. 2020). Furthermore, the current therapies available for treating depression are frequently associated with a range of undesirable side effects (gastric, hepatotoxicity, loss of appetite and weight) (Campos et al. 2005); according to the WHO in 2019, more than 1000 billion Dollars are spent per year on the purchase of antidepressants. In addition, the delayed therapeutic effects associated with patients’ resistant to these treatments highlight the urgent need to find new and more effective alternative treatments (Pham and Gardier, 2019).

Interestingly, animal models have been extensively used to demonstrate that chronic restriction is related to chronic exposure to stress, enabling the understanding of MDD and the screening of novel therapeutic agents (Seo et al. 2017).There is growing interest in traditional medicine, as several arguments suggest that medicinal plants are relatively inexhaustible reservoirs of medicines and are more compatible with the human body, generally having few undesirable side effects (El-Wahab et al. 2013). Medicinal plants are still being studied on a daily basis, with the aim of improving treatment for various diseases (Mpondo et al. 2017). This is the case with traditional Chinese medicine, which uses Honokiol, a bioactive polyphenolic compound extracted from the *Magnolia officinalis*, which has been shown to have ameliorating effects on depression (Poivre and Duez, 2017).

*Momordica foetida* (Cucurbitaceae) is a perennial climbing herb with tendrils and cream coloured flowers. Its characteristic fruit is bright orange with prickles (Acquaviva et al., 2013). It is widely distributed in tropical South and West Africa (Oloyede and Aluko, 2012). In many countries across Africa, *M. foetida*is is used by local people to treat a number of illnesses such as headache, cough, intestinal disorders, toothache, skin problems (Getachew, 2015). Previous phytochemical studies resulted in the isolation of cucurbitane triterpenoids from the leaf extract, alkaloids and glycosides from the whole plant and the identification of sitosteryl glycoside, 5,25-stigmastadien-3ß-yl-glucoside and 1ß-hydroxyfriedel-6-en-3-un (Froelich et al. 2007). Moreover, previous studies have shown potential antidepressant-like effect in *M. foetida*methanol extract (Kada et al.2022). However, the specific beneficial effects of *M. foetida* on MDD have not yet been demonstrated. The aim of the present study was therefore to evaluate the antidepressant effect of *M. foetida* aqueous lyophilisate on rat.

## Materials and methods

### Plant

Stems and leaves of *Momordica foetida* were collected during the rainy season (May, 2022) in the West region of Cameroon, precisely in Menoua division, at the locality of Fokoué and authenticated at the National Herbarium of Cameroon in Yaoundé, in comparison with material from SCA 877, herbarium collection N^º^ 33417/HNC. These parts of plant were shade dried for several days and transformed into powder which was later used for extract preparation.

The aqueous mixture of *M. foetida* was obtained by macerating 400 g of powder in 4 L of distilled water for 24 h (Ngoupaye et al., 2020) and then lyophilized at 0^0^ C at the Institute for Medical research and study of Medecinal Plants (IMPM) in Yaoundé, Cameroon. The extraction yield was 10.8 %, determined using the method described by Pahaye et al. (2017). The lyophilizate was administered *p. o* at a volume of 10 mL/kg body weight.

### Animals

Adult Wistar albino rats of both sexes, aged 2–3 months with an average weight of 120–140 g were used. The animals were raised at room temperature and subjected to a natural light-dark cycle in the animal house of the Department of Animal Biology, University of Dschang, Cameroon; and were separated in subgroup of 4 males and 3 females per group before and during the treatment to avoid mating (Yassi et al. 2023). They were given water and food *ad libitum*. Experiments were carried out in accordance with NIH-Care and use of laboratory animals’ manual, and Directives 2010/63/EU for animal experiments. Efforts were also made to minimize animal suffering as much as possible.

## Chemical

Prozac (Fluoxétine) (LILLY, France) was administered at the dose of 15 mg/kg (Ngoupaye et al., 2020).

## Treatment

A total of 42 Wistar rats were divided into 06 groups of 07 animals each, and treated as follows:

▪ Group 1: received only distilled water (10 mL/kg, *p.o*) and corresponded the neutral control,
▪ Group 2: received distilled water (10 mL/kg, *p.o*) and was subjected to chronic restriction and corresponded the negative control,
▪ Group 3: received fluoxetine as the reference substance (15 mg/kg, *p.o*) and was subjected to chronic restriction and corresponded as a positive control,
▪ Group 4: received the aqueous lyophilisate (25 mg/kg, *p.o*) and was subjected to chronic restriction,
▪ Group 5: received the aqueous lyophilisate (50 mg/kg, *p.o*) and was subjected to chronic restriction,
▪ Group 6: received the aqueous lyophilisate (75 mg/kg, *p.o*) and was subjected to chronic restriction.

Animals were subjected to chronic restriction 1 hour after receiving different treatment.

## Evaluation of the effect of *M. foetida’s* aqueous lyophilisate on MDD

### Induction of MDD

The rats were exposed to a stressor induced by 14 days of chronic restriction, allowing for an in-depth investigation of the neurological, behavioural and physiological changes associated with depression. Chronic restriction consisted of placing the animals of the various groups, with the exception of the neutral control group, individually in a propylene tube (6 cm wide and 10 cm long) for 2 h every day for 14 days. The wall of the propylene tube was perforated by a few holes 1 cm in diameter near the animal's head to ensure proper ventilation. The animals were deprived of food, water and movement throughout the daily restriction period.

To avoid familiarisation of the animal in the propylene tube, the 2 h daily restriction sessions were carried out according to the schedule in Table 1 (Ngoupaye et al., 2022).

**Table 1:** Table illustrating the restraint stress model. This schedule was repeated until day 14.


Schedule		
Day 1	2 h	0 h
Day 2	1 h	1 h
Day 3	0 h	2 h
Day 4	2 h	0 h
Day 5	1 h	1 h
Day 6	0 h	2 h
Day 7	2 h	0 h

**Fig. 1:**
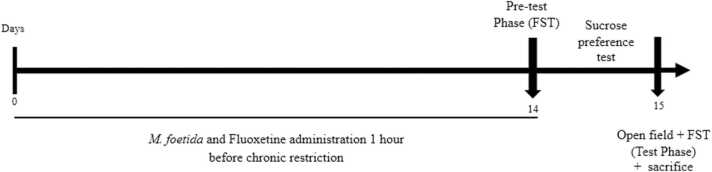
Schematic representation of drug administration. FST: Forced swimming test.

Animals were returned to their original cages during the break. The schedule was repeated for the next seven days. All animals in the neutral control group were treated with distilled water daily for 14 days (Ngoupaye et al., 2022). The behavioural tests used to assess the depressive behaviour of the animals included the forced swim test, the sucrose preference test and the open field test. Fig. 1 below represents the sequencing of the different tests and drug administration.

### The forced swim test

The forced swim test is a commonly used paradigm for the depression trials, allowing for the assessment of despair in animals and the evaluation of the antidepressant potential of certain substances. The method described by (Porsolt et al. 1977) was used in the present study. The test consisted of two phases: an induction or pre-test phase and a test phase. During the induction phase, the rats were placed individually in a Plexiglas cylindrical tank (50 cm height, 18 cm diameter) filled with water (23–25°C) up to 30 cm high for 15 min. 24 h following this pre-test phase, they were placed in the same glass cylinder for 5 min, constituting the test phase. The animals' movements were recorded and the immobility and swimming time was measured for the last 4 min. Immobility was defined as the absence of movement for at least 2 seconds, or only the minimal movements required to keep the animal's nose above the water.

### Sucrose preference test

This test evaluates anhedonia, a hallmark symptom of MDD, characterized by a loss of pleasure. The rats were tested on their preference for sucrose for 24 hours. Two bottles were placed in the animals' cages, each containing two different solutions. One containing only drinking water and the other containing a 5 % sucrose solution. The bottles were then changed positions after 12 hours to prevent the animals from becoming habituated. The bottles were weighed before and after being placed in the cage for every 12 h of observation time and the value was converted into mL in order to measure the amount of liquid consumed, allowing to assess the anhedonia of these animal. Subsequently, sucrose preference was calculated by the following formula: Preference = sucrose (mL) / (sucrose (mL) + water (mL) (Ngoupaye et al., 2018).

## The open field test

In order to assess the effects of the different treatments on locomotor and exploratory activity, the animals were subjected to an open field test. The open field test is a closed square enclosure, illuminated at its centre and allowing the animal inside neither to escape nor to hide. The device used was a 40 cm square and divided into 16 equal squares of 10 cm^2^. The walls were 19 cm high (Yassi et al. 2023). Each animal was placed individually in the centre of the arena and allowed to explore it freely for 5 min. Parameters indicating the activity of each animal were recorded, namely: 'crossing' (number of squares crossed by the animal) and 'rearing' (number of times the animal stood on its hind legs while leaning on the wall of the open field). After five minutes of observation, each animal was returned to a cage and the device was cleaned with ethyl alcohol (70°).

### Animal sacrifice and collection of organs

One hour after the last behavioural test, the animals were sacrificed by decapitation. The hippocampus was collected under aseptic conditions and placed in an Eppendorf tube, then stored at low temperature (-20ºC).

### Preparation of the homogenate

The left hippocampus and prefrontal cortex were ground at 10 % (w/v) in a porcelain mortar. Each sample was ground with 0.1 M phosphate buffer containing 1 % Triton-100X (pH 7.4). The homogenates obtained were centrifuged (3000 rpm) for 15 min at room temperature and the supernatant obtained was used to assay oxidative stress parameters (Foutsop et al. 2023).

The right hippocampus was ground in the ratio of 5 mg organ to 100 µL grinding solution (37 % HCl + Butanol). The mixture was then centrifuged (2000 rpm) for 10 min. 500 µL of the supernatant was added to 1250 µL of heptane and 155 µl of 0.1 M HCl. This mixture was vigorously shaken for 10 min, then centrifuged (2000 rpm) for 10 min. The top organic phase was discarded, while the bottom aqueous phase was recovered to serve as a homogenate for the determination of monoamines level (Schumfjf et al. 1974).

### Evaluation of protein content

The protein assay was performed according to the recommendations of a commercially available protein kit with ready-to-use reagents (Dutch Diagnostics, Germany). Following the kit's recommendations, 10 µL of homogenate was introduced into 500 µL of biuret reagent. The mixture was incubated for 5 min at room temperature. The supernatant was pipetted and the absorbance read at 550 nm using a BIORAD spectrophotometer (Smart Spec 3000, USA) against the blank. Protein concentration was expressed in mg/mL.

### Determination of malondialdehyde levels

Malondialdehyde (MDA) reacts under heat with thiobarbituric acid (TBA) to produce a pinkish chromophore. 250 µL of 20 % tricloroacetic acid (TCA), 500 µl of 0.67 % thiobarbituric acid, 250 µl of homogenate and 10 µL of 0.1 % butylatedhydroxytoluene (BHT) were introduced into a test tube. The blank tube contained all of the above, with the exception of the homogenate. All tubes were then sealed and incubated for 10 min at 90°C. After cooling, the tubes were centrifuged at 3000 rpm for 10 min at room temperature. The supernatant was pipetted and read at absorbance of 532 nm using a BIORAD spectrophotometer (Smart Spec 3000, USA) against the blank and the concentration of MDA in the sample was expressed in nmol/mg of tissue (Dimo et al. 2006).

### Determination of reduced glutathione levels

Glutathione (GSH) is capable of reacting with Ellman's reagent to form a yellow coloration. A volume of 1500 µL of Ellman's reagent (0.1 mM of 5,5′-dithiol bis-2-nitrobenzoic acid in 0.3 M phosphate buffer added to 1 % sodium citrate solution) was introduced into test tubes containing 100 µL phosphate buffer (PBS) and 100 µl of homogenate. Next, the mixture was incubated for 1 h at room temperature. The mixture was read using a BIORAD spectrophotometer (Smart Spec 3000, USA) at an absorbance of 412 nm against the blank. GSH concentration of the sample was expressed in nmol/mg of tissue (Foutsop et al. 2023).

### Assessment of catalase

Catalase is an antioxidant enzyme that catalyses the conversion of hydrogen peroxide (H_2_O_2_) into water (H_2_O) and oxygen (O_2_). When dichromate is in the presence of acetic acid and H_2_O_2,_ it is reduced to chromic acetate, which can be measured colorimetrically (Dimo et al. 2006). PBS (375 µL) and homogenate (25 µL) were first added to a tube·H_2_O_2_ 50 mM (100 µL) was then added and 60 seconds later, potassium dichromate (5 %) + acetic acid solution (volume) was added to stop the reaction. The tube was stirred and sealed before being incubated for 10 min (temperature). At the end of incubation, the tubes were cooled under running water and the contents of each tube pipetted and read using a BIORAD spectrophotometer (Smart Spec 3000, USA) against the blank. The absorbance was read at 570 nm. The enzymatic activity of catalase was expressed in µmol of H_2_O_2_ decomposed/min/mg of protein, based on the calibration curve of H_2_O_2._

### Assessment of superoxide dismutase

This assay is based on the ability of superoxide dismutase (SOD) to delay the auto-oxidation of adrenaline to adenochrome in a basic medium. Carbonate buffer pH= 10.2 (1660 µL) and homogenate (140 µL) were added to the tube. Next, 0.3 mM adrenaline (200 µl) was added to the tube and the stopwatch started. Three readings were taken with a BIORAD spectrophotometer (Smart Spec 3000, USA) against the blank, at 60, 120 and 180 seconds respectively at absorbance 480 nm. SOD activity was expressed in units per mg of protein (Dimo et al. 2006).

### Determination of nitric oxide levels

A volume of 125 µL of homogenate and 125 µL of Griess reagent were added to test tubes. The whole mixture was left in the dark for 5 minutes. The optical densities were then read using a BIORAD spectrophotometer (Smart Spec 3000, USA) against the blank, at 540 nm. The level of nitric oxide was determined by a calibration curve established from the different concentrations of nitrite (Na_2_ NO) (Taiwe et al. 2015).

### Determination of tissue levels of serotonin

In the presence of HCl, serotonin is degraded to give a compound that reacts with O-phthaldialdehyde (OPT), giving a chromophore whose colour intensity is proportional to the level of serotonin. In a test tube, 200 µL of homogenate was mixed with 250 µL of OPT. The tube was then boiled in a water bath at 100°C for 10 min. After cooling under running water, the absorbance was read at 470 nm using a BIORAD spectrophotometer (Smart Spec 3000, USA) against the blank. The serotonin concentration of the sample was expressed in ng/mg of protein (Schumfjf et al. 1974).

### Determination of Tissue levels of dopamine and noradrenaline

Dopamine and noradrenaline are oxidised in the presence of iodine to give chromophores that absorb at different wavelengths. The intensity of the staining is proportional to the concentration of dopamine and noradrenaline in the tissue. The reagents are introduced in the following order: 200 µL of homogenate, 50 µL of HCl 0.4 M and 100 µL of sodium acetate buffer. Next, 100 µL of 0.1 M iodine was added to the reaction medium. 2 min later, the reaction was stopped by introducing 100 µL of sodium sulphite into the medium. 90 seconds later, 100 µL of 10 M acetic acid was added to the medium. The tube was then sealed and heated in a water bath at 100°C for 6 min. After cooling with running water, the absorbance was read at 375 nm for dopamine and 485 nm for noradrenaline using a BIORAD spectrophotometer (Smart Spec 3000, USA) against the blank. The concentration of dopamine or noradrenaline in the sample was expressed in ng/mg of protein (Schumfjf et al. 1974).

## Quantitative phytochemistry of bioactive secondary metabolites and anti-oxidant power of *Momordica* foetida aqueous lyophilisate

### Quantification of total phenols

The total phenolic content was determined by the method described by Ramde-Tiendrebeogo et al. (2012). The reagent consists of a mixture of phosphotungstic acid (H_3_ PW O_1240_) and phosphomolybdic acid (H_3_PMo O_1240_). On oxidation, the phenols are reduced to a mixture of blue oxides of tungsten and molybdenum. These blue pigments have a maximum absorption that varies according to the qualitative and/or quantitative composition of the phenolic mixtures in addition to the pH of the solutions, generally obtained by adding sodium carbonate. The reaction mixture in this test consisted of 20 µL of extracts (2 mg/mL), 100 µL of Folin-Ciocalteu reagent (diluted 10-fold in water) and 80 µL of a 20 % sodium carbonate solution. The mixture was stirred and incubated in a water bath at 20°C for 30 min, then the absorbance was measured with a spectrophotometer at 765 nm. The extracts were replaced by distilled water in the blank tubes. A calibration curve was drawn using gallic acid (the concentration of gallic acid varied from 0.015 to 2 mg/mL). The results were expressed as milligram equivalents of gallic acid per gram of extract.

### Quantification of total flavonoids

The total flavonoid content of the extracts was determined using the aluminium chloride colorimetric method (Chang et al. 2002). A volume of 100 µL of extract (2 mg/mL) was mixed with 50 µL of aluminium chloride (1.2 %), then 50 µL of potassium acetate (120 mM) was added. The mixture was incubated for 30 min at room temperature and the absorbance was measured with a spectrophotometer at 415 nm. Total flavonoid content was calculated using the quercetin calibration curve (quercetin concentration ranged from 0.015 to 2 mg/mL) and results were expressed as milligram quercetin equivalent per gram extract.

### Quantification of total tannins

Tannin content was determined using the Folin-Ciocalteu method as described by Govindappa et al. (2011). Briefly, the reaction mixture in this test consisted of 100 µL of extracts (2 mg/mL), 500 µL of Folin-Ciocalteu reagent (diluted 10-fold in water), 1000 µL of a 35 % sodium carbonate solution and 8.4 mL of distilled water. The mixture was stirred and incubated at room temperature for 30 min, then the absorbance was measured with a spectrophotometer at 700 nm. The extracts were replaced with distilled water in the blank tubes. A calibration curve was drawn using tannic acid (the concentration of tannic acid varied from 100, 200, 300, 400, 500 µg/ mL). The results were expressed as milligram equivalents of tannic acid per gram of extract.

### Study of antioxidant activity using the DPPH (2,2-Diphenyl-2-picrylhydrazyl) test

The DPPH assay of samples was evaluated as described by Mensor et al. (2001). In each well of a 96-well plate, 20 µL of methanol was introduced in the last seven rows. This was followed by the introduction of 20 µL of the methanolic solutions of the test samples (2 mg/mL) into the first two wells of each column (4 columns were used for one sample) and successive 2-fold serial dilutions were made in the other wells, maintaining the volume at 20 µL. 180 µL of methanolic solution of DPPH (0.08 mg/mL) was again introduced into each well of the first three columns while 180 µL of methanol was introduced into each well of the fourth column. Plates containing 200 µL of final solution per well were incubated for 30 min in the dark at room temperature. At the end of the incubation, the optical densities were read with a Microplate Reader (FLUOstar Omega microplate reader) at 517 nm and converted into antioxidant activity percentages. Vitamin C (L-ascorbic acid) was used as a positive control. Three replicates were performed for each sample. The antioxidant activity percentages for each sample were calculated according to the following formula:

$$\mathrm{antioxidantactivity}\left(\mathrm{in}\%\right)=\frac{\mathrm{DPPHAbsorbance}\left(\mathrm{AssayAbsorbance}-\mathrm{BlankAbsorbance}\right)}{\mathrm{DPPHAbsorbance}}X100$$

The different percentages of antioxidant activity were used to determine the ECs_50_ (the sample concentration able to trap 50 % of DPPH) (Yassaet al. 2008).

### Study of antioxidant activity using the FRAP method (*Ferric reducing antioxidant power*)

The reducing power of the samples was determined according to the protocol described by Benzie and Strain (1996). The FRAP reagent was prepared by mixing a buffer solution of sodium acetate (300 mM, pH 3.6), a solution of 2,4,6-tris (2-pyridyl) −1,3,5-s-triazine TPTZ (10 mM) and a solution of FeCl_3_ in the proportions 10:1:1. A volume of 5 µL of sample (2 mg/mL) was mixed with 95 µL of FRAP reagent. The mixture was incubated for 30 min at 37°C in the dark. After incubation, the optical density was read with a Microplate Reader (FLUOstar Omega Microplate Reader) at 593 nm. Vitamin C was used as a positive control. The antioxidant capacity of the sample was calculated from the calibration curve of the FeSO_4_ solution (the number of moles of the FeSO_4_ solution ranging from 156.25 µmol to 10,000 µmol) and expressed as the micromole equivalent of FeSO_4_ per gram of sample.

### Statistical analysis

The results were analysed using Graph PadPrism software, version 5.03. The parameters measured during the behavioural tests and the assays were expressed as the mean ± standard error on the mean (SEM). Values were compared using one-way and two-way analysis of variance (ANOVA) tests, and where differences existed, the Newman Keuls and Bonferoni multiple comparison tests were used to separate means. Values were considered statistically significant at p < 0.05.

## Results

### Effect of *M. foetida* aqueous lyophilisate on major depression

#### Effect of *M. foetida* aqueous lyophilisate on major depression assessed in the Forced Swimming Test (FST)

**Fig. 2:**
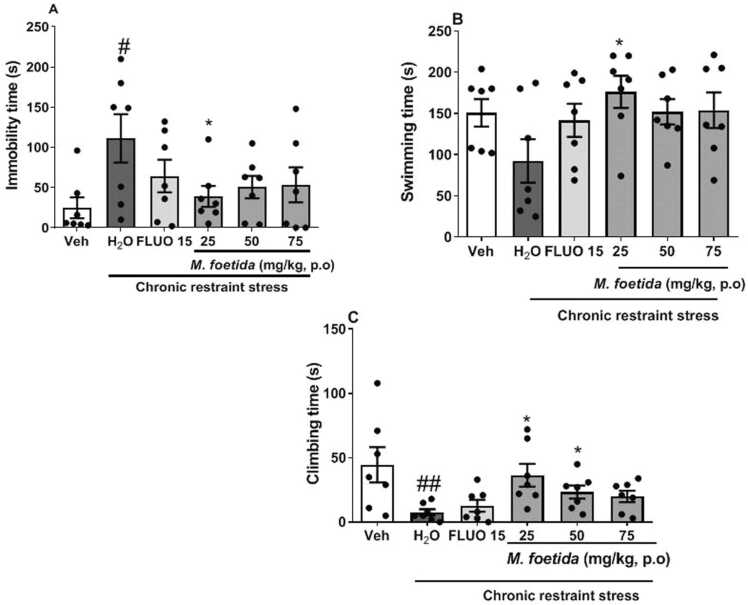
Effects of *M. foetida* aqueous lyophilisate on MDD assessed in the forced swimming test. A= Immobility time; B= Swimming time; C= Climbing time. Data expressed as mean ± SEM; n = 7; one-way ANOVA followed by Newman-keuls post-test; Veh= Neutral control; H_2_O= Distilled water: negative control; FLUO = Fluoxetine (15 mg/kg).

Fig. 2a show the effect of *M. foetida* aqueous lyophilisate on the immobility time recorded in the FST.

A stress effect was observed as the negative control showed an increase in immobility time compared to the vehicle [F (2,20) = 4.853, p = 0.0089]. Animals treated with the aqueous lyophilisate showed a tendency to reduce immobility time at the dose 50 mg/kg [F (2,20) = 2.843, p = 0.0845] and a significant reduction at the dose 25 mg/kg [F (2,20) = 4.187, p = 0.0321] compared to the negative control.

Fig. 2b depicts the effect of *M. foetida* aqueous lyophilisate on swimming time in FST.

The negative control showed a tendency to decrease swimming time compared to the vehicle [F (2,20) = 2.739, p = 0.0915]. The administration of *M. foetida* aqueous lyophilisate showed an increase in the swimming time compared to the negative control with an increasing tendency at the dose 50 mg/kg [F (2,20) = 3.063, p = 0.0716] and a marked effect at the dose 25 mg/kg [F (2,20) = 4.193, p = 0.0320] (Fig. 2b).

The negative control showed a significant decrease in climbing time compared to the vehicle [F (2,20) = 6.885, p = 0.0060]. Animals treated with *M. foetida* aqueous lyophilisate at the dose 25 mg/kg [F (2,20) = 5.121, p = 0.0173] and 50 mg/kg [F (2,20) = 4.391, p = 0.0280] showed a significant increase in the climbing time compared to the negative control. The dose 75 mg/kg showed a tendency to increase the climbing time [F (2,20) = 3.376, p = 0.0569] (Fig. 2c).

### Effect of *M. foetida* aqueous lyophilisate on major depression assessed in the open field test

**Fig. 3:**
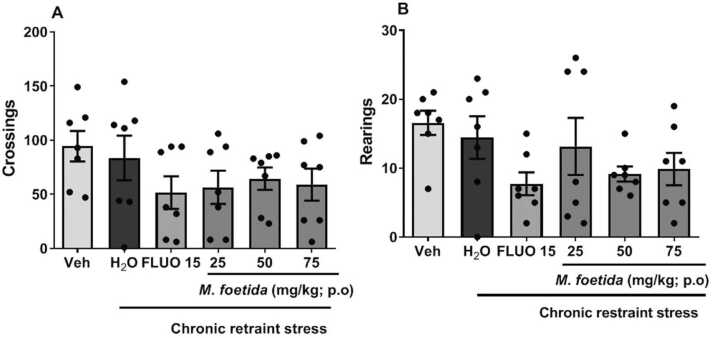
Effects of *M. foetida* aqueous lyophilisate on the number of lines crossed. A=Number of lines crossed; B=Number of rearing; Data expressed as mean ± SEM; n = 7; One-way ANOVA followed by Newman-Keuls post-test; Veh= Neutral control; H_2_O= Distilled water: negative control; FLUO = Fluoxetine (15 mg/kg).

Fig. 3a shows the effect of *M. foetida* aqueous lyophilisate on the number of lines crossed by the animals following various treatments.

There was no significant decrease in the number of lines crossed between the groups compared with the negative control [F (5,41) = 1.220, p = 0.3196] (Fig. 3a), nor in rearing between the groups compared with the negative control [F (5,41) = 1.801, p = 0.1375] (Fig. 3b).

### Effect of *M. foetida* aqueous lyophilisate on the sucrose preference test (TS)

**Fig. 4:**
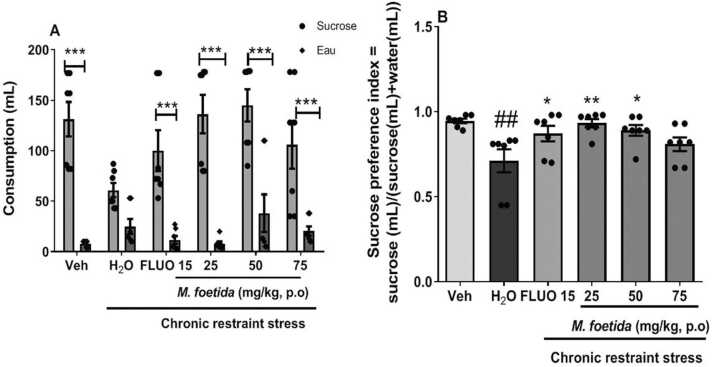
Effects of *M. foetida* aqueous lyophilisate on the sucrose preference test. A=Sucrose and water consumption; B=Preference index. Data expressed as mean ± SEM; n = 7; * p < 0.05; * * p < 0.01; * ** p < 0.001 when compared to the distilled water group; ## p < 0.01 when compared to the neutral control group; Two-way ANOVA followed by Bonferroni test (A) and one-way ANOVA followed by Newman-Keuls post-test (B); Veh= Neutral control; H_2_O= Distilled water: negative control; FLUO = Fluoxetine (15 mg/kg).

Fig. 4a shows the effect of *M. foetida* aqueous lyophilisate on water and sucrose consumption. There was a stress-induced effect as evidenced by the significant decrease in the sucrose consumption and an increase in water consumption in the negative control compare to the vehicle.

Animals treated with the aqueous lyophilisate of *M. foetida* showed an increase in sucrose consumption and a decrease in water consumption compared to the negative control, at all doses group [F (1,72) = 136.2, p < 0.0001].

Fig. 4b depicts the effect of *M. foetida* aqueous lyophilisate on the sucrose preference index. The negative control showed a decrease in the sucrose preference index compared to the vehicle [F (2,20) = 11.19, p = 0.0007]. Fluoxetine showed an increase trend of the sucrose preference index compared with the negative control [F (2,20) = 2.907, p = 0.0805]. Animals treated with *M. Foetida* aqueous lyophilisate increased the sucrose preference index compared to the negative control, at 25 mg/kg [F (2,20) = 5. 053, p = 0.0181], 50 mg/kg [F (2,20) = 4.858, p = 0.0206] and 75 mg/kg [F (2,20) = 1.206, p = 0.3225].

### Effect of *M. foetida* aqueous lyophilisate on oxidative stress parameters in the hippocampus

**Fig. 5:**
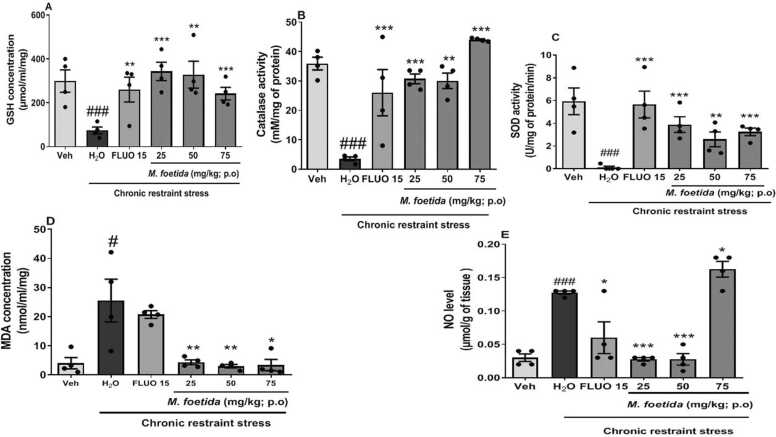
Effects of *M. foetida* aqueous lyophilisate in the hippocampus on the different parameters of oxidative stress induced by chronic restriction. Data expressed as mean ± SEM; n = 4; * p < 0.05; * * p < 0.01; * ** p < 0.001 when compared to the distilled water group; ### p < 0.001 and ## p < 0.01 when compared to the neutral control group; One-way ANOVA followed by Newman-Keuls post-test; Veh= Neutral control; H_2_O= Distilled water: negative control; FLUO = Fluoxetine (15 mg/kg).

Fig. 5a shows the effect of *M. foetida* aqueous lyophilisate on the level of reduced glutathione (GSH).

The negative control showed a decrease in the level of GSH, compared to the vehicle [F (2,11) = 16.40, p = 0.0001]. Fluoxetine increased this concentration compared to the negative control, [F (2,11) = 9.377, p = 0.0063]. Animals treated with *M. foetida* aqueous lyophilisate showed an increase in the level of GSH compared to the negative control, at the doses 25 mg/kg [F (2,11) = 32.37, p < 0.0001], 50 mg/kg [F (2,11) = 15. 25, p = 0.0013], and 75 mg/kg [F (2,11) = 21.30, p = 0.0004] with a marked effect at the dose 25 mg/kg.

Fig. 5b shows the effect of *M. foetida* aqueous lyophilisate on catalase activity.

The negative control showed a decrease in catalase activity compared to the vehicle [F (2,11) = 72.13, p < 0.0001]. Fluoxetine, increased catalase activity compared to the negative control [F (2,11) = 72.13, p < 0.0001]. Animals treated with *M. foetida* aqueous lyophilisate showed an increase in catalase activity compared to the negative control at the doses 25 mg/kg [F (2,11) = 188.8, p < 0.0001 50 mg/kg [F (2,11) = 8.023, p = 0.0100] and 75 mg/kg [F (2,11) = 91.95, p < 0.0001].

Fig. 5c shows the effect of *M. foetida* aqueous lyophilisate on superoxide dismutase (SOD) activity.

The negative control showed a decrease in SOD activity compared to the vehicle [F (2,11) = 24.20, p = 0.0002]. Fluoxetine increased SOD activity compared to the negative control [F (2,11) = 21.65, p = 0.0004]. Animals treated with *M. foetida*aqueous lyophilisate showed an increase in SOD activity compared to the negative control, at the doses 25 mg/kg [F (2,11) = 28.72, p = 0.0001],.50 mg/kg [F (2,11) = 13. 61, p = 0.0019], and 75 mg/kg [F (2,11) = 72.13, p < 0.0001]

Fig. 5d shows the effect of *M. foetida* aqueous lyophilisate on malondialdehyde (MDA) level.

The negative control showed an increase in MDA compared to the vehicle [F (2,11) = 6.413, p = 0.0186].

Animals treated with *M.foetida* aqueous lyophilisate showed a decrease in MDA level compared to the negative control, at the doses 25 mg/kg [F (2,11) = 8.112, p = 0.0097], 50 mg/kg [F (2,11) = 9. 285, p = 0.0065], 75 mg/kg [F (2,11) = 7.973, p = 0.0102].

Fig. 5e shows the effect of *M. foetida* aqueous lyophilisate on nitric oxide (NO) level.

The negative control showed an increase in NO level compared to the vehicle [F (2,11) = 130.4, p < 0.0001]. Fluoxetine showed a decrease in NO concentration compared to the vehicle [F (2,11) = 7.867, p = 0.0106].

Animals treated with *M. foetida* aqueous lyophilisate showed a decrease in NO level compared to the negative control, at the doses 25 mg/kg [F (2,11) = 212. 9, p < 0.0001], 50 mg/kg [F (2,11) = 86.62, p < 0.0001]. However, at the dose 75 mg/kg [F (2,11) = 4.292, p = 0.0491], there was an increase in NO levels.

### Effect of *M. foetida* aqueous lyophilisate on monoamines in the hippocampus

**Fig. 6:**
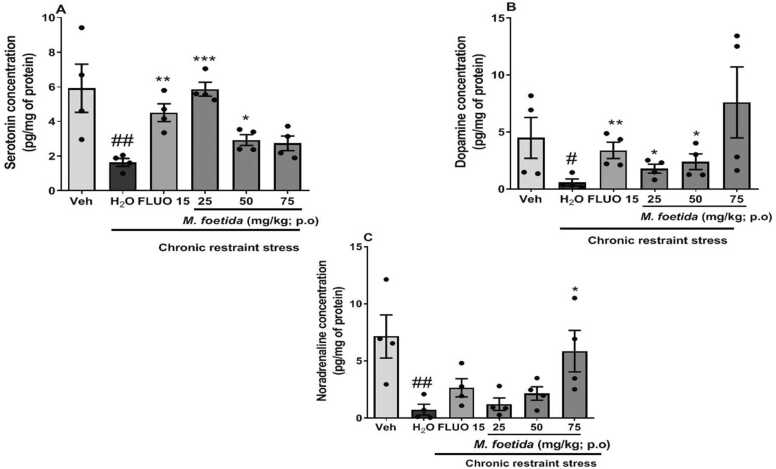
effect of *M. foetida* aqueous lyophilisate in the hippocampus on monoamines. Data expressed as mean ± SEM; n = 4; * p < 0.05; * * p < 0.01; * ** p < 0.001 when compared to the distilled water group; # p < 0.05 and ## p < 0.01 when compared to the neutral control group; One-way ANOVA followed by Newman-Keuls post-test; Veh= Neutral control; H_2_O= Distilled water: negative control; FLUO = Fluoxetine (15 mg/kg).

Fig. 6a shows the effect of *M. foetida* aqueous lyophilisate on serotonin level.

Animals from the negative control showed a decrease in serotonin level compared to the vehicle [F (2,11) = 8.401, p = 0.0087].

Animals treated with *M. foetida* aqueous lyophilisate at the doses 25 mg/kg [F (2,11) = 36.56, p < 0.0001] and 50 mg/kg [F (2,11) = 6.696, p = 0.0165] showed an increase in serotonin concentration compared to the negative control with a marked effect at the dose 25 mg/kg.

Fig. 6b shows the effect of *M. foetida* aqueous lyophilisate on dopamine level.

The negative control showed a decrease in dopamine level compared to the vehicle [F (2,11) = 4.505, p = 0.0441]. Fluoxetine showed an increase in this concentration compared to the negative control [F (2,11) = 11.51, p = 0.0033].

Animals treated with *M. foetida* aqueous lyophilisate showed an increase in dopamine level compared with the negative control, at 25 mg/kg [F (2,11) = 4. 392, p = 0.0467] and 50 mg/kg [F (2,11) = 5.075, p = 0.0334].

Fig. 6c shows the effect of *M. foetida* aqueous lyophilisate on noradrenaline level.

The negative control showed a decrease in the noradrenaline level compared to the vehicle [F (2,11) = 7.144, p = 0.0052].

Animals treated with *M. foetida* aqueous lyophilisate did not significantly affect the noradrenaline levels, except the dose 75 mg/kg [F (3,15) = 6.278, p = 0.0196] which showed a significant increase compared to the negative control.

### Antioxidant power of *M. foetida* aqueous lyophilisate: Study of antioxidant activity using the DPPH test and the FRAP method

Table 2 shows that the aqueous lyophilisate of *M. foetida* increased antioxidant activity compared to the positive control. This activity increased from 8.29 ± 0.13 µg/mL in the positive control to 8145.16 ± 0.15 µg/mL in the *M. foetida* aqueous lyophilisate.

**Table 2:** effect of *M. foetida* aqueous lyophilizateon the antioxidant activity.

Sample	Test to DPPH ECs_50_ (µg/mL)	FRAP method (µmol/mL FeSO_4_/g)
*M. foetida*	8145.16 ± 0.15	14.19 ± 0.42
Vitamine C	8.29 ± 0.13	160.42 ± 0.35

**Legend:** DDPH = 2,2-Diphenyl-2-picrylhydrazyl; FRAP= Ferric reducing antioxidant power

The *M. foetida* aqueous lyophilisate demonstrated a lower reducing strength compared to the positive control, with values decreasing from 160.42 ± 0.35µmol/mL in the positive control to 14.19 ± 0.42µmol/mL in *M. foetida* aqueous lyophilisate.

### Quantitative phytochemical composition of *M. foetida* aqueous lyophilisate

Table 3 shows the qualitative and quantitative phytochemistry of different bioactive secondary metabolites.

**Table 3:** Qualitative and quantitative phytochemist composition of *M. foetida* aqueous lyophilisate.

Sample	TPT (mg EAG/g of lyophilisate)	TFT (mg EQ/g of lyophilisate)	TTT (mg EAT/g of lyophilisate)
*M. foetida*	3.75 ± 0.16	1.45 ± 0.32	1.15 ± 0.11
Butylhydroxytolene	426.85 ± 0.68	61.89 ± 0.97	52.43 ± 0.31

**Legend:** TPT = Total Phenol Weigh; TFT = Total Flavonoid Weigh; TTT = Total Tannins Weigh.

EAG = Equivalent of Gallic Acid; EQ = Equivalent Quercetin; EAT = Equivalent of Tannic Acid.

## Discussion

This study was designed to evaluate the antidepressant effect of the aqueous lyophilisate of *M. foetida* following exposure to chronic stress. As such, several tests were conducted, including the forced swimming test (FST), the Open field (OFT) and the sucrose preference test (SPT). The FST is used to assess depressive-like behaviours and to identify of potential antidepressant drugs such as selective serotonin reuptake inhibitors (SSRIs), other types of tricyclic antidepressants, monoamine oxidase inhibitors and many others (Ngoupaye et al. 2014). This test is based on the hypothesis that immobility reflects a measure of behavioural hopelessness which is a depression-like behaviour (Cryan et al. 2005). Indeed, in FST, rats are forced to swim in a narrow space with no escape. Thus, after an initial period of vigorous activity, they move only to keep their heads above water. SSRIs are known to reduce immobility following chronic administration in rats (Overstre et al., 2004). The aqueous lyophilisate of *M. foetida* (25 mg/kg, 50 mg/kg and 75 mg/kg) reduced the immobility time observed in FST, suggesting that *M. foetida’s* aqueous lyophilisate has a potential antidepressant effect. Antidepressants that enhance serotonergic neurotransmission are known to result in prolonged swimming time in the FST, while those that increase noradrenergic neurotransmission lead to longer climbing time (Cryan et al. 2005). *M. foetida’s* aqueous lyophilisate at the dose 25 mg/kg increased swimming time in the forced swim test, similar to the effect of fluoxetine (15 mg/kg), a SSRI, suggesting that *M. foetida* aqueous lyophilisate’s activity may be mediated through the serotonergic pathway.

Some antidepressants are known to reduce immobility in the FST but are however not effective in treating depression. It is therefore recommended to perform locomotor activity tests in addition to FST to rule out that baseline activity level is not the determining factor in this model (Bal 2012; Ngoupaye et al. 2014). To rule out the possibility that the observed effects of *M. foetida* in the FST were due to psychostimulant effects (which also reduce immobility time in the FST but increase locomotor activity), an Open Field Test (OFT) was conducted to assess locomotor activity in rodents (Sousa et al. 2004). The results obtained showed that there was no significant difference in locomotor activity expressed by the number of lines crossed by animals treated with the aqueous lyophilisate of *M. foetida* and or fluoxetine, just like animals of the negative control group who did not receive any treatment, compared with healthy animals. This result therefore confirms the antidepressant effects of the aqueous lyophilisate of *M. foetida*.

Depression is generally accompanied by anhedonia, which is one of its characteristic symptoms (Matraszek-Gawron et al., 2023). In this study, this symptom was highlighted by the SPT. Indeed, a decrease in preference for sucrose indicates anhedonia, which reflects the loss of the ability to feel pleasure (Moore et al. 2018). Just like fluoxetine, *M. foetida’s* aqueous lyophilisate at all doses significantly increases sucrose consumption compared to water. These assertions were confirmed in the sucrose consumption index which was significantly elevated for the animals which received the lyophilisate at the doses 25 mg/kg and 50 mg/kg compared to the negative control, demonstrating the ability of the aqueous lyophilisate of *M. foetida* to counteract the reduction in reward-seeking behaviour that tends to occur in chronically restricted and untreated animals.

Depression has multiple causes, with involvement from various brain regions (such as the hippocampus and prefrontal cortex), as well as complex neural pathways (Trifu et al. 2020). At the molecular level, oxidative stress is a key contributor to the development of depression, influencing its progression through various mechanisms (Bhatt et al. 2020). Mitochondrial function is closely linked to oxidative stress and overproduction of reactive oxygen species through mitochondrial dysfunction or reduction in antioxidant defence can cause oxidative damages, particularly in the brain where overproduction of the latter is implicated in many psychiatric disorders, such as depression (Khan et al. 2023). Indeed, the brain is particularly prone to oxidative stress because it contains a large quantity of transition metals and polyunsaturated fatty acids that provide a substrate for lipid peroxidation, in addition to its high rate of oxygen consumption and limited antioxidant defences (Ng et al. 2008). Depression is characterised by lipid peroxidation, as indicated by higher levels of malondialdehyde (MDA) in negative control animals. Thus, chronic restriction resulted in an increase in parameters related to oxidative stress and this can be observed in animals of the negative control group, which show a significant increase in MDA and nitric oxide levels in the hippocampus. However, the aqueous lyophilisate of *M. foetida* at all doses significantly reduced the levels of these two parameters in the hippocampus excepted for NO where the dose 75 mg/kg showed an increase in NO concentration.

In addition, as with fluoxetine, the doses 25 mg/kg 50 mg/kg and 75 mg/kg showed a significant increase in tissue levels of reduced glutathione (GSH), superoxide dismutase (SOD) and catalase activity compared to the negative control group. The lyophilisate demonstrates antioxidant properties by neutralizing free radicals. Indeed, this can be explained by the presence of polyphenols in the lyophilisate which are known to have antioxidant activity (Xia et al. 2021). Another group of bioactive compounds present in the lyophilisate are flavonoids and animal studies have demonstrated that flavonoids possess an antidepressant-like property via interactions with oxidative pathways and antioxidant systems (Yi et al. 2010). The lyophilisate therefore reduces the behavioural symptoms of depression and modulates the biological parameters associated with depression in this model.

The DPPH (diphenylpicrylhydrazyl) assay is a widely used method in the analysis of oxidative activity (Molyneux, 2003). It has been widely used to determine free radical scavenging capacity, with tannins and flavonoids being very effective radical scavengers (Amarowicz et al., 2004). Indeed, flavonoids contain conjugated ring structures and hydroxyl groups that have the potential to function as antioxidants by scavenging superoxide anion, singlet oxygen, peroxidised lipids, and stabilising free radicals involved in oxidative processes by hydrogenation or complexation with oxidising species (Wafa et al. 2016). Antioxidant molecules such as flavonoids and tannins have been shown to reduce and bleach DPPH due to their ability to give up hydrogen (Bougandoura and Bendimerad, 2012). These molecules in the lyophilisate are probably responsible for its antioxidant activity. In addition, flavonoids and tannins have increased ferric reducing power. Tannins have the ability to chelate metal ions such as Fe (II) and interfere with one of the reaction steps in the Fenton reaction, thereby delaying oxidation (Wafa et al. 2016). The reducing power of the *M. foetida* aqueous lyophilisate is also thought to be due to the presence of the hydroxyl group in the phenolic compounds, which can act as an electron donor.

Excessive production of reactive oxygen species can lead to the destruction of phospholipids and reduce the flexibility of the cell membrane. These changes can affect the density and function of serotonin, dopamine and noradrenaline, which have important roles in the pathophysiology of depression (Bal et al. 2012). Indeed, the increase in ROS and reactive nitrogen species resulting from high levels of oxidative stress severely damages deoxyribonucleic acid nucleotides and alters the production of these neurotransmitters (Hussain et al. 2019). Depression would therefore result from the dysfunction of monoaminergic transmission systems, following a decrease in the levels of neurotransmitters involved such as serotonin, dopamine and noradrenaline (Patillon, 2014). Expectedly, decreased levels of catecholamines, altered densities and functions of serotonergic and catecholaminergic receptors, and decreased binding of catecholamines to their sites of action may all have roles in the development of depression (Maes and Meltzer, 1995). Current pharmacotherapies for depression act on monoamine neurotransmitters, namely SSRIs (selective serotonin reuptake inhibitors) such as fluoxetine which prevent the reuptake of serotonin and MAOIs which inhibit monoamine oxidase (Berton and Nestler, 2006). Of interest, fluoxetine through its active metabolite norfluoxetine, which is activated by P450 (CYP2D6), exerts its effects by blocking the reuptake of serotonin in the presynaptic serotonergic neuron’s terminal. However, it has minimal activity on noradrenergic reuptake (Robertson et al., 2019, Cao et al., 2019). In this study, it was shown that chronic restriction is responsible for the depressive behaviour observed in FST and this is due to the decrease in monoamine levels, mainly serotonin, dopamine and noradrenaline. Taken together, the results show that, like fluoxetine, the lyophilisate reduces depressive behaviour via the serotonergic and dopaminergic pathways, as evidenced by the significant increase in serotonin and dopamine levels at doses of 25 mg/kg and 50 mg/kg in the hippocampus. It should be noted that *M. foetida* significantly increased the forced swimming time, indicating the involvement of the serotonergic pathway, and also prevented a reduction in the sucrose preference index, indicating that it preserves the state of pleasure and inhibits anhedonia. The increase in serotonin and dopamine levels confirms the experimental results obtained in the behavioural tests. At the same doses, the lyophilisate reduced immobility time and attenuated anhedonia. The lyophilisate produces effects similar to conventional antidepressants like fluoxetine, by inhibiting serotonin reuptake and thereby increasing the duration of serotonin activity in the brain. The antidepressant effect of *M. foetida’ s* aqueous lyophilisate is associated with monoaminergic and antioxidant activity. These effects are reflected in improved mood and relieved depressive symptoms.

## Conclusion

This work led us to the conclusion that the aqueous lyophilisate of *M. foetida* has an antidepressant effect. This effect is mediated via the modulation of oxidative stress, the involvement of the serotonergic and dopaminergic neurotransmission through the release of serotonin and dopamine, supported by the reduction of immobility time and increase in swimming time assessed in the FST and the increase in sucrose consumption.

## Ethics approval

Animal experiments were carried out in accordance with the international principles of laboratory animal protection (NIH Publication 8023, revised 1996), and use of laboratory animals’ manual, and Directives 2010/63/EU for animal experiments. Efforts have been made to minimize animal suffering as much as possible.

## Code availability

Not applicable.

## Consent to Participate

Not applicable.

## **Consent for publication**

Not applicable.

## Funding

No specific grant, fund from funding agencies were receiving during this research.

## **Conflict of interest and Compliance with ethical standards**

The authors have non-financial interests to disclose. Animal experiments were carried out in accordance with the international principles of laboratory animal protection (NIH Publication 8023, revised 1996), and use of laboratory animals’ manual, and Directives 2010/63/EU for animal experiments. Efforts have been made to minimize animal suffering as much as possible.

## CRediT authorship contribution statement

**Kom Tatiana Diebo:** Writing – original draft, Investigation, Conceptualization. **Ngoufack Steve Brunel Kenfack:** Methodology. **Adassi Blesdel Maxwell:** Writing – original draft. **Ngo Bum Elisabeth:** Supervision, Project administration. **Yassi Francis Bray:** Methodology, Investigation. **Ngoupaye Gwladys Temkou:** Writing – review & editing, Visualization, Validation, Supervision, Resources, Formal analysis, Data curation, Conceptualization. **Foutsop Aurelien Fossueh:** Writing – original draft.

## Declaration of Competing Interest

The authors declare no conflict of interest.
